# Mutational and clinical analysis of the *ENG* gene in patients with pulmonary arterial hypertension

**DOI:** 10.1186/s12863-016-0384-3

**Published:** 2016-06-04

**Authors:** Guillermo Pousada, Adolfo Baloira, Diego Fontán, Marta Núñez, Diana Valverde

**Affiliations:** Department Biochemistry, Genetics and Immunology, Faculty of Biology, University of Vigo, As Lagoas Marcosende S/N, 36310 Vigo, Spain; Instituto de Investigación Biomédica de Vigo (IBIV), Vigo, Spain; Complexo Hospitalario Universitario de Pontevedra, Servicio de neumología, Pontevedra, Spain

**Keywords:** Pulmonary arterial hypertension, *ENG* gene, Mutational analysis, Functional study, Genotype-phenotype correlation

## Abstract

**Background:**

Pulmonary arterial hypertension (PAH) is a rare vascular disorder characterized by a capillary wedge pressure ≤ 15 mmHg and a mean pulmonary arterial pressure ≥ 25 mmHg at rest. PAH can be idiopathic, heritable or associated with other conditions. The aim of this study was to analyze the Endoglin (*ENG*) gene and assess the influence of the c.572G > A (p.G191D) mutation in patients with idiopathic or associated PAH. The correlation between the pathogenic mutations and clinical and functional parameters was further analyzed.

**Results:**

Sixteen different changes in the *ENG* gene were found in 44 out of 57 patients. After in silico analysis, we classified eight mutations as pathogenic in 16 of patients. The c.572G>A (p.G191D) variation was observed in ten patients, and the analysis for the splicing process using hybrid minigenes, with *pSPL3* vector to assess splicing alterations, do not generate a new transcript. Age at diagnosis (*p* = 0.049) and the 6-min walking test (*p* = 0.041) exhibited statistically significant differences between carriers and non-carriers of pathogenic mutations. Patients with pathogenic mutations exhibited disease symptoms 8 years before non-carriers. Five patients with pathogenic mutations were carriers of another mutation in the *BMPR2* or *ACVRL1* genes.

**Conclusions:**

We present a series of PAH patients with mutations in the *ENG* gene, some of them not previously described, exhibiting clinical and hemodynamic alterations suggesting that the presence of these mutations may be associated with the severity of the disease. Moreover, genetic analysis in patients with PAH may be of clinical relevance and indicates the complexity of the genetic background.

**Graphical Abstract:**
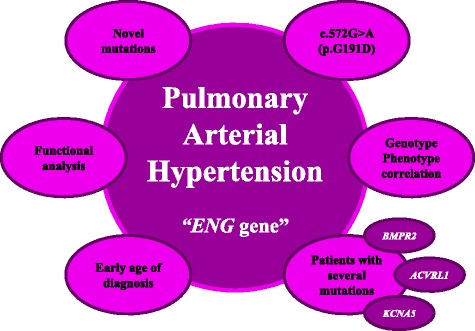
The role of *ENG* gene in patients with Pulmonary Arterial Hypertension.

## Background

Pulmonary arterial hypertension (PAH; OMIM *#*178600; ORPHA 422) is a severe disease affecting small pulmonary arteries that results in progressive remodeling leading to elevated pulmonary vascular resistance and right ventricular failure [1]. PAH can be idiopathic (IPAH), heritable (HPAH) or associated with other conditions (APAH) [2]. PAH is characterized by a capillary wedge pressure ≤ 15 mmHg and mean pulmonary arterial pressure ≥ 25 mmHg at rest [1, 2]. Symptoms of PAH are mixed but include dyspnea, syncope and chest pain. Eventually, the disease in these patients leads to right-sided heart failure and death [1]. The main pathologic changes associated with increased pulmonary vascular resistance are thrombus development, thickened intima, proliferation of smooth muscles cells, and growth of plexiform lesions in pulmonary vessels [3]. The estimated incidence is approximately 2–5 cases per million per year [3], and the gender ratio is 1.7:1 female vs male [4, 5]. Without treatment, the disease progresses to right ventricular failure and death within 3 years of diagnosis [6].

Heterozygous germline mutations in the bone morphogenetic protein type 2 receptor (*BMPR2;* MIM #600799) have been identified in approximately 10 to 40 % of IPAH patients without a reported familial history of the disease and in over 80 % of patients with HPAH [4, 7–9]. PAH patients with *BMPR2* mutations are reported to develop more severe disease, are less likely to respond to treatment and are diagnosed approximately 10 years earlier than non-carriers [10]. In a few PAH patients, mutations in other genes participating in the *BMPR2* signaling pathway have been reported, including Endoglin, also known as CD105, (*ENG;* MIM #601284) [11]. *ENG* gene mutations are less common than *BMPR2* gene mutations in patients with PAH. Accordingly, a more complicated genetic background has been proposed for PAH [7].

The *ENG* gene encodes a type I integral membrane glycoprotein receptor that is a member of the Transforming growth factor beta (TGF-β) signaling superfamily. This receptor is expressed on proliferating vascular endothelial cells and in other cell types associated with cardiovascular system and controls diverse cellular processes, including cell differentiation, proliferation, angiogenesis, inflammation, and wound healings and has been linked to psoriatic skin, inflamed synovial arthritis, vascular injury, tumor vessels and apoptosis in embryonic and mature tissues [12–15]. The human *ENG* gene is located on chromosome 9q33-34 [7, 13, 14], and the encoded protein exhibits an extracellular domain, hydrophobic transmembrane domain and a cytosolic domain. The extracellular domain contains 561 amino acids and is the largest of the domains [13]. This gene is implicated in hereditary hemorrhagic telangiectasia (HHT) type 1, an autosomal dominant syndrome characterized by vascular dysplasia. Mutations found in the *ENG* gene are an important factor for the development of HHT and may contribute to PAH in some HHT patients due to the gene’s function as a TGF-β receptor [7, 13–16]. Mutations in this gene are frequently identified in HHT but are uncommon in PAH patients [4, 15, 17].

The potential role of *ENG* gene in patients with PAH remains unknown. To analyze its impact in patients with IPAH and APAH, we analyzed the coding region and intronic junctions of this gene and try to associate hemodynamic and clinical characteristics between carriers and non-carriers of *ENG* mutations. To evaluate the effect of *ENG* mutations on clinical outcomes of PAH, the phenotypical characteristic of carriers of missense mutations and carriers of mutations that alter the splicing in this gene were compared.

## Methods

### Patients and samples

As described previously [8], patients with idiopathic or associated PAH (group 1 of the classification of Nice) [18] treated in our clinic were included in this study. All patients are included in the CHUVI DNA Biobank (Biobanco del Complejo Hospitalario Universitario de Vigo). Patients signed an informed consent and the Autonomic Ethics Committee approved the study (Comité Autonómico de Ética da Investigación de Galicia-CAEI de Galicia).

In all cases, cardiac catheterization was performed using the latest consensus diagnostic criteria of the ERS-ESC (European Respiratory Society-European Society of Cardiology) [19]. PAH was considered idiopathic after the exclusion of any of the possible causes associated with the disease. Clinical histories included use of drugs, especially appetite suppressants, and screening for connective tissue diseases and hepatic disease. The study included serology for Human immunodeficiency virus (HIV), autoimmunity, thoracic tomography computerized scan (TC scan) and echocardiography. Patients with PAH that could be related to chronic lung disease were excluded. Fifty-five healthy individuals were used as controls.

### Mutational analysis

Venous blood was collected from patients and healthy volunteers to extract genomic DNA using the FlexiGene DNA Kit (Qiagen, Hilden, Germany) according to the manufacturer’s protocol.

Amplification of the *ENG* gene was performed with 50 ng of genomic DNA from each patient and control. We amplified the exon regions and intronic junctions and did not analyzed changes in other regions for this study. The primers use to amplified this region by PCR (Polymerase chain reaction) were described by Gallione et al [20], with minor modifications (Table 1). The PCR mix was GoTaq® Green Master Mix (Promega Corporation, Madison, Wisconsin, USA), which contained Taq DNA polymerase, dNTPs, MgCl_2_ and reaction buffer. A second independent PCR and sequencing reaction in both the forward and reverse strands was performed to check for the detected mutations. PCR was performed in an MJ MiniTM Gradient Thermal Cycler (Bio-Rad, Hercules, California, USA). Electrophoresis on a 2 % agarose gel containing ethidium bromide was performed to confirmed PCR products in a Sub-Cell GT (Bio-Rad, Hercules, California, USA). HyperLadder IV-V (New England Biolabs, Ipswich, Massachusetts, USA) was used as the molecular weight marker. PCR fragments were purified using the ExoSAP-IT kit (USB Corporation, Cleveland, Ohio, USA) and sequenced with the BigDye Terminator version 3.1 Cycle Sequencing Kit (Applied Biosystems, Carlsbad, California, USA). The sequencing reactions were precipitated and analyzed on an ABI PRISM 3100 genetic analyzer (Applied Biosystems, Carlsbad, California, USA).

**Table 1:** Primers used to amplify the *ENG* gene

EXON	PRIMERS		SIZE	Tª
	Forward 5’ → 3’	Reverse 5’ → 3’		
1	ACTGGACACAGGATAAGGCCCAGC	AATACTTGGGGCCTGGTCCGTG	180 bp	62 °C
2	CACCTTATTCTCACCTGGCCTCTT	CTGCCTTGGAGCTTCCTCTGAG	249 bp	61 °C
3	GGGTGGCACAACCTATACAAAT	CAGAGATGGACAGTAGGGACCT	269 bp	60 °C
4	CTACATGGGATAGAGAGGGCAC	TTCCTCCTGAGCAGTATCATGAG	277 bp	55 °C
5	TGAGGGAAGGGACTGAGGTGC	GTGGGGACTAGTGTCAGGGGC	238 bp	63 °C
6	GGCCTGTCCGCTTCAGTGTT	GTTTTGTGTCCCGGGAGCTG	203 bp	58 °C
7	CCCCCTGTTCTGCCTCTCTC	CTGATCCAAGGGAGGGGAAG	256 bp	63 °C
8	ACACATATCACACAGTGACCAGC	CTAGGGGAGGAACCAGATGTC	253 bp	55 °C
9	CTCCTGATGGTGCCCCTCTCTTC	TTGTCTTGTGTTCTGAGCCCCTG	296 bp	60 °C
10	CTGCAGGGGCTCAGAACACA	GGCCAGGTGGGTTAGCACG	212 bp	61 °C
11	ATTGACCAAGTCTCCCTCCC	GAAAGGCGGAGAGGAAGTTC	211 bp	61 °C
12	GGTGGGGTGAAGAGCAGCTG	GACCTGGAAGCTCCCACTTGAA	359 bp	58 °C
13	GAGTAAACCTGGAAGCCGC	GCCACTAGAACAAACCCGAG	154 bp	55 °C
14 A	CCAGCACAACAGGGTAGGGGAT	CTCAGAGGCTTCACTGGGCTCC	255 bp	61 °C
14 B	AGGACCCTGACCTCCGCC	CTCTCCTGCTGGGCGAGC	198 bp	63 °C

Sequence data were aligned to the reference Ensembl cDNA sequence ENSG00000106991 for the *ENG* gene and examined for sequence variations. To align and compare sequences in different organisms we use the Basic Local Alignment Search Tool (BLAST) software. Polyphen-2 (available at http://genetics.bwh.harvard.edu/pph/) characterize an amino acid substitution as “benign”, “possibly damaging” or “probably damaging” [21], Pmut (available at http://mmb2.pcb.ub.es:8080/PMut/) provides a binary prediction of “neutral” or “pathologic” [22], Sort Intolerant from Tolerant (*SIFT*) (available at http://sift.jcvi.org) predict whether a change is “tolerated” or “damaging” [23] and MutationTaster2 software (available at http://www.mutationtaster.org/) characterize an amino acid substitution as “polymorphism” or “disease causing” [24] computer algorithms were used to predict whether missense variants were pathological. A brief explanation for these software programs is provided in Pousada et al [8]. The mutations were classified as pathogenic if the score were equal or greater than two.

NNSplice (available at http://fruitfly.org:9005/seq_tools/splice.html), NetGene2 (available at http://www.cbs.dtu.dk/services/NetGene2/), Splice View (available at http://zeus2.itb.cnr.it/~webgene/wwwspliceview_ex.html) and HSF Human (available at http://www.umd.be/HSF/) were used to predict whether changes could affect, create or eliminate donor/acceptor splice sites [8]. The mutations were classified as pathogenic if the score were equal or greater than two.

### Minigene constructions and expression

For the c.572G>A (p.G191D) change, we amplified the exon and 200 bp of intronic junctions from the control DNA with High Fidelity Phusion polymerase (Finnzymes, Espoo, Finland) to obtain the wild-type (WT). The amplification conditions were as follows: 98 °C for 30 s, 35 cycles of 98 °C for 10 s, 60 °C for 30 s, 72 °C for 30 s and, finally, 72 °C for 7 min. The amplified fragments were digested and cloned into the XhoI/NheI restriction sites (Nzytech, Lisbon, Portugal) using T4 DNA ligase (New England Biolabs, Ipswich, Massachusetts, USA) in the Exon Trapping Expression Vector p.SPL3 (Invitrogen, San Diego, California, USA). The c.572G>A (p.G191D) construct was generated by site-directed mutagenesis. The primers used for mutagenesis were designed using QuikChange Primer Design (Agilent Technologies, Santa Clara, California, USA). The forward and reverse primers were 5'-gccaggacatggaccgcacgctcga-3' and 5'-tcgagcgtgcggtccatgtcctggc-3', respectively. All constructs were confirmed by direct sequencing.

COS-7 cells (from kidney of *Cercopithecus aethiops*) were transfected in duplicated by the minigene constructs. Lipofectamine 2000 reagents (Invitrogen, San Diego, CA, USA) were used according to the manufacturer’s instructions. RNA extraction was performed using the Nucleic Acid and Protein Purification kit (NucleoSpin RNA II, Macherey-Nagel, Düren, Germany). RNA was subjected to reverse transcription using the GeneAmp Gold RNA PCR Core Kit (Applied Biosystems, Carlsbad, California). cDNA was amplified and PCR products were sequenced in both senses.

### Statistical analysis

A non-parametric test (U Mann-Whitney) was used for comparisons between patients and controls and this study is exploratory. The Chi-square test was used to compare genotypes with clinical and hemodynamic variables and variables were categorized according to the best cut off point by ROC curve. Analyses were supported by the statistical package SPSS v19 for Microsoft and we considered differences statistically significant at values <0.05. Values were expressed as the mean ± SD (standard deviation).

## Results

### Description of the cohort

**Fig. 1:**
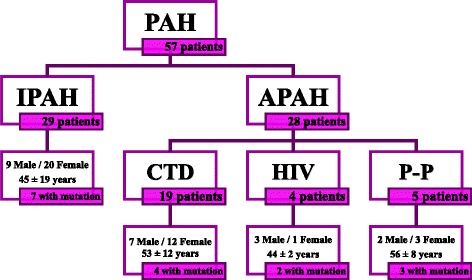
Nature of the patient cohort. This figure describes the patients involved in this analysis separated by PAH type, the proportion of mutation carriers in the study, the female to male proportion and the mean age at diagnosis. PAH: Pulmonary Arterial Hypertension; IPAH: Idiopathic Pulmonary Arterial Hypertension; Associated Pulmonary Arterial Hypertension; CTD: connective tissue disease; HIV: Human Immunodeficiency virus; P-P: Porto-pulmonary hypertension

This cohort has been described previously by our group [8, 25] and included 57 unrelated PAH patients (29 idiopathic, 19 associated with connective tissue disease, four related to HIV and five porto-pulmonary) (Fig. 1). Samples from PAH patients who agreed to participate in the study were collected between 2008 and 2014. At the time of diagnosis, eight patients were functional class (FC) I, 20 patients were FC II, 25 patients were FC III and four were FC IV. The clinical features of the patients are presented in Table 2.

**Table 2:** Clinical features and hemodynamic parameters of patients

Clinical features and hemodynamic parameters	Total patients	Carriers of pathogenic mutations^a^		Carriers of p.G191N mutation^b^	
		Clinical data	*p*-value	Clinical data	*p*-value
Number	57	16	----------	10	----------
Gender	21 M/36 F	6 M/10 F	n.s	2 M/8 F	n.s
Age at diagnosis (years)	49 ± 15	41 ± 16	0.040	39 ± 18	0.035
mPaP (mmHg)	49 ± 14	48 ± 11	n.s	45 ± 12	n.s
sPaP (mmHg)	70 ± 19	75 ± 16	n.s	67 ± 18	n.s
PVR (mmHg.l^−1^.m^−1^)	8.2 ± 3.5	8.6 ± 3.2	n.s	8.3 ± 0.9	n.s
CI (l.m^−1^.m^−2^)	2.6 ± 0.6	2.5 ± 0.7	n.s	2.1 ± 0.5	0.049
6MWT (m)	445 ± 139	323 ± 162	0.040	457 ± 172	n.s
PAH types	29 IPAH/28 APAH	7 IPAH/9 APAH	n.s	8 IPAH/2 APAH	0.040

Values are expressed as the mean ± standard deviation; *F* female, *M* male, *mPaP* mean pulmonary artery pressure, *sPaP* systolic pulmonary artery pressure, *PVR* pulmonary vascular resistance, *CI* cardiac index, *6MWT* 6 min walking test, *IPAH* idiopathic pulmonary arterial hypertension, *APAH* associated pulmonary arterial hypertension

^a^We have compared clinical features and hemodynamic parameters between patients with mutations in *ENG* gene and patients without mutations

^b^We have compared clinical features and hemodynamic parameters between patients with p.G191N variation in *ENG* gene and patients without mutations

In the present study, 55 controls from the general population without a familial history of PAH were included to determine the frequency of the mutations detected in the *ENG* gene. Samples were kindly provided by the Complexo Hospitalario Universitario de Vigo (Vigo, Spain).

### Mutational study of the *ENG* gene

**Fig. 2:**
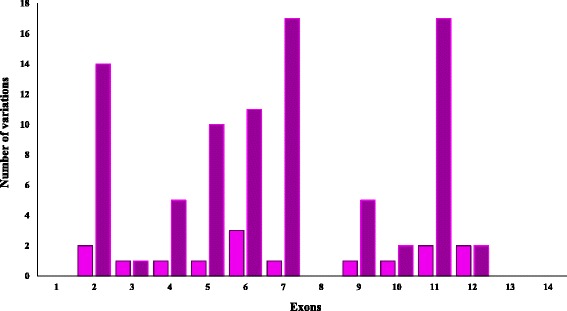
Mutational frequency of each of the exons of the *ENG* gene. The pink color indicates the number of different mutations found in each exon, and the purple color indicates the total of mutations found in each exon for the *ENG* gene

We found 15 variants of the *ENG* gene in 44 out of 57 patients. We detected eight different variations first described here and seven changes that have been described elsewhere. The vast majority of these changes were detected in amplicon 7 and 11 (Fig. 2), but we detected the exons 6 and 12 as hotspots for pathogenic mutations. The novel variations did not appear in 55 analyzed controls (110 chromosomes). After an exhaustive in silico analysis, we could identify 8 variations as pathogenic mutations.

**Fig. 3:**
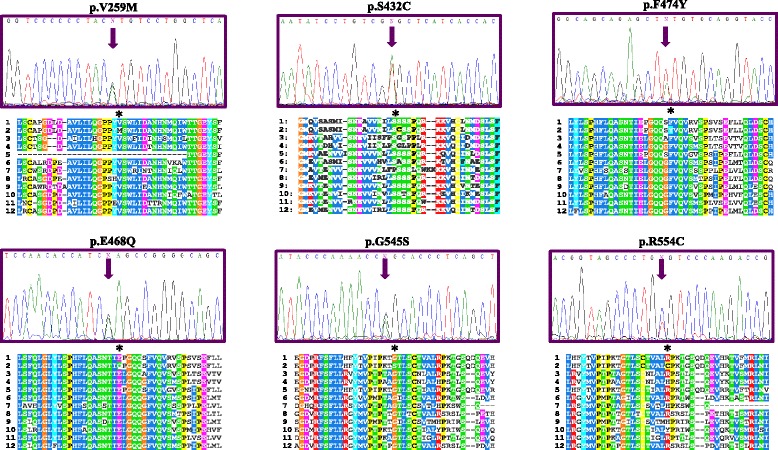
Representative sequence electropherograms for the missense variations for the *ENG* gene in PAH patients with their orthologs. 1: *Homo sapiens* (sp|P17813#1); 2: *Homo sapiens* mutated (sp|P17813#1); 3: *Mus musculus* (sp|Q63961#1); 4: *Rattus norvegicus* (sp|Q6Q3E8#1); 5: *Macaca mulatta* (sp|F7BB68#1); 6: *Sus scrofa* (sp|P37176#1); 7: *Oryctolagus cuniculus* (sp|G1SSF2#1); 8: *Canis familiaris* (sp|F1P847#1); 9: *Bos taurus* (sp|Q1RMV1#1); 10: *Equus caballus* (sp|F6 W046#1); 11: *Loxodonta africana* (sp|G3SR82#1); 12: *Ailuropoda melanoleuca* (sp|G1 M9D6#1)

Missense variations were analyzed with different software programs (PolyPhen, Pmut, Sift and Mutation Taster) to predict their pathogenicity and the impact on the disease. We classified the mutation as potentially pathogenic if two or more programs classified it as pathogenic (Table 3). These analyses classified five missense mutations as pathogenic mutations; however, c.572G>A (p.G191D) has been classified as polymorphism by other studies [26–28]. Figure 3 presents the amino acid conservation involved in these missense changes. We observed that the wild-type residues in the p.(S432C) and p.(R554C) mutations are not perfectly conserved between *Homo sapiens* (human) and ten other species, but are conserved amongst some of the species analyzed.

**Table 3:** Missense changes found in the coding region of the *ENG* gene and their classification according to computer algorithms (*PolyPhen-2*, *Pmut*, *SIFT* and *MutationTaster2*)

Classification of missense variations found in the coding region								
Exon	Nucleotide change	Amino acid change	Times detected	PolyPhen-2	Pmut	Sift	Mutation Taster	Score
Exon 5	c.572G>A	p.(G191D)	10	Probably Damaging	Pathological	Damaging	Disease causing	4
Exon 6	c.775G>A	p.(V259M)	3	Probably Damaging	Neutral	Damaging	Polymorphism	2
Exon 10	c.1295A>T	p.(S432C)	2	Probably Damaging	Pathological	Tolerated	Polymorphism	2
Exon 11	c.1402G>C	p.(E468Q)	12	Probably Damaging	Neutral	Tolerated	Polymorphism	1
Exon 11	c.1421T>A	p.(F474Y)	6	Probably Damaging	Neutral	Tolerated	Polymorphism	1
Exon 12	c.1633G>A	p.(G545S)	1	Probably Damaging	Pathological	Tolerated	Disease causing	3
Exon 12	c.1660C>A	p.(R554C)	1	Probably Damaging	Pathological	Tolerated	Polymorphism	2

These results are considered damaging if the score is equal or greater than two

For the six intronic changes detected, only a duplication (c.991 + 21_991 + 26dupCCTCCC) had been described previously as a polymorphism. This duplication was detected in 35 % of patients included in this study but also in 8 % of controls.

We used other algorithms (*NNSplice*, *NetGene2*, *Splice View* and *HSF Human*) to predict whether these missense, synonymous and intronic changes could affect donor/acceptor splice sites. We classified the mutation as potentially pathogenic if two or more programs classified it as pathogenic (Table 4).

**Table 4:** Results from the four different bioinformatic programs used to predict the effect of missense, synonymous and intronic changes on the splicing process in the *ENG* gene (*NNSplice*, *NetGene2*, *Splice View* and *HSF Human*)

Sequence variants	Reference	Genotype frequency	NNSplice	NetGene2	Splice View	HSF Human	Score
c.207G>A (p.(L69L))	rs11545664	G: 89 % A: 11 %	Neutral	Neutral	Neutral	A new acceptor site is created	1
c.219+25G>T	This study	----------	Neutral	Neutral	Neutral	Neutral	0
c.360+56T>A	This study	----------	Neutral	Score for the main donor site decreases from 93 to 89	Neutral	A new acceptor site is created	2
c.498G>A (p.(Q166Q))	Pousada et al [8]	G: 100 % A: 0 %	Neutral	Score for the main donor site decreases from 90 to 87	A new donor site is created	Score for the main acceptor site decrease from 82 to 53	3
c.572G>A (p.(G191D))	Rs41322046 (Lesca et al [27])	G: 100 % A: 0 %	Neutral	Score for the main acceptor site increase from 18 to 19	Neutral	Neutral	1
c.775G>A (p.(V259M))	This study	----------	Neutral	Score for the main acceptor site increase from 35 to 37	Neutral	A new acceptor site is created	2
c.817+17T>A	This study	----------	Neutral	Score for the main donor site decreases from 100 to 99	Neutral	Score for the main acceptor site decrease from 82 to 78	2
c.817+23G>A	This study	----------	Neutral	Neutral	Neutral	Neutral	0
c.991+21_991+26dupCCTCCC	rs148063362	WT: 74 % DUP: 26 %	Neutral	Neutral	Neutral	Neutral	0
c.1272+6A>T	This study	----------	Neutral	Neutral	A new donor site is created	Score for the main acceptor site decrease from 65 to 37	2
c.1295A>T (p.(S432C))	This study	----------	Neutral	Score for the main donor site decreases from 74 to 54	Neutral	Score for the main acceptor site decrease from 76 to 72	2
c.1402G>C (p.(E468Q))	rs370554511	G: 100 % C: 0 %	Neutral	Neutral	The WT consensus sequence is not recognized	Score for the main acceptor site increase from 70 to 80	1
c.1421 T>A (p.(F474Y))	This study	----------	Neutral	Neutral	Neutral	Score for the main acceptor site decrease from 87 to 85	1
c.1633G>A (p.(G545S))	rs1428896669 (Pfarr et al [7])	G: 100 % A: 0 %	Neutral	Neutral	Neutral	A new acceptor site is created	1
c.1660C>A (p.(R554C))	COSM1105417	C: 100 % A: 0 %	Neutral	Score for the main donor site decreases from 69 to 67	Neutral	A new acceptor site is created	2

These results are considered positive if the score is equal or greater than two. The Genotype frequency values were for 1000 Genome Project. For novel mutations, described in this study, no genotype data were available

These pathogenic mutations were detected in 16 patients, four mutations were missense (except c.572G > A (p.G191D), as has been classified as polymorphism by other authors), one synonymous and three were located in the intronic region. Of these patients, seven were classified as IPAH and in nine as APAH.

### Study of the c.572G>A (p.G191D) change

**Fig. 4:**
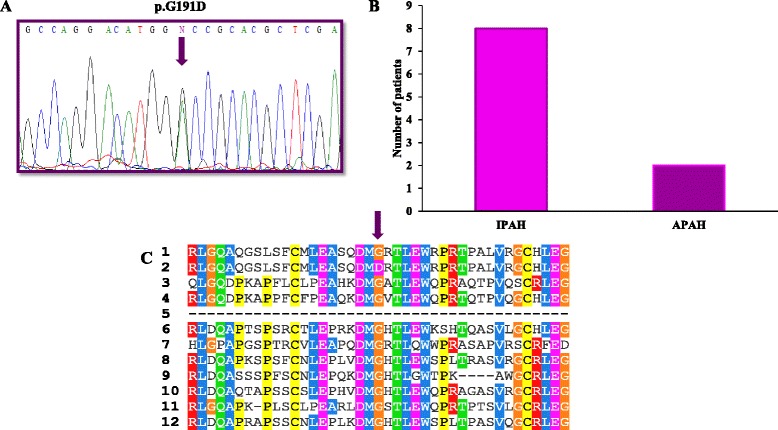
**a** Representative sequence electropherograms for the c.572G > A (p.(D191G)) mutation for the *ENG* gene in PAH patients. **b** Different orthologs for this mutation. **c** Mutational frequency for this pathogenic mutation in IPAH and APAH patients. 1: *Homo sapiens* (sp|P17813#1); 2: *Homo sapiens* mutated (sp|P17813#1); 3: *Mus musculus* (sp|Q63961#1); 4: *Rattus norvegicus* (sp|Q6Q3E8#1); 5: *Macaca mulatta* (sp|F7BB68#1); 6: *Sus scrofa* (sp|P37176#1); 7: *Oryctolagus cuniculus* (sp|G1SSF2#1); 8: *Canis familiaris* (sp|F1P847#1); 9: *Bos taurus* (sp|Q1RMV1#1); 10: *Equus caballus* (sp|F6W046#1); 11: *Loxodonta africana* (sp|G3SR82#1); 12: *Ailuropoda melanoleuca* (sp|G1M9D6#1)

**Fig. 5:**
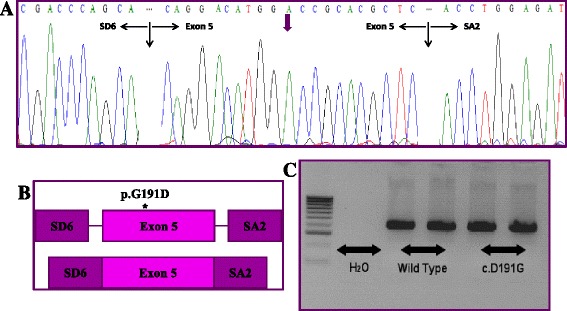
In vitro splicing assay for the c.572G > A (p.G191D) change identified in the *ENG* gene. **a** Electropherogram of the transcript obtained indicates the molecular characterization of the effect of the studied variant. **b** Graphical representation of the effect of p.G191D change in mRNA processing. **c** Electrophoresis of wild-type and mutant construction. SDS and SA2: pSPL3 vector exons, where the inserts to study are cloned

This change c.572G>A (p.G191D) was found in ten patients included in this study and was more frequent in IPAH than in patients with APAH. This change was not detected in 110 control alleles (*p* = 0.001). In patients, the G allele frequency was 0.909 (90 %). Allele A was not detected in controls. This change was not in Hardy-Weinberg Equilibrium (H-WE) in patients (*p* = 0.617), in contrast to the controls (*p* < 0.001). BLAST software indicated that the G amino acid (glycine) is an evolutionarily conserved residue (Fig. 4). We checked for alterations in the splicing process using hybrid minigenes for this gene in comparison to the wild type sequence. The mutant construct did not generate a new transcript (Fig. 5). All experiments were performed in duplicate.

### Association with clinical features and hemodynamic parameters

**Fig. 6:**
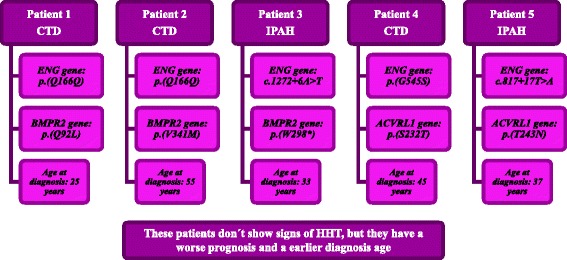
Mutational analysis of patients with multiple pathogenic mutations in analyzed genes

None of the clinical features or hemodynamic parameters exhibited statistically significant differences, except for age at diagnosis (*p* = 0.040) and the 6-min walking test (*p* = 0.040). Patients with pathogenic mutations in *ENG* gene exhibited disease symptoms 8 years earlier and were diagnosed earlier than patients with a negative mutational screening for *ENG*, *BMPR2*, *ACVRL1* (Activin A Receptor Type II-Like 1) and *KCNA5* (Potassium voltage-gated channel, shaker-related subfamily, member 5) genes (Table 2). However, five patients with *ENG* pathogenic mutations were also carriers for another mutation in the *BMPR2* or *ACVRL1* genes (Fig. 6) as described by Pousada et al [8]. When removing these patients for statistical analysis, only age at diagnosis was significantly different (mean 9 years early, *p* = 0.040).

The c.572G>A (p.G191D) change was associated with an early age at diagnosis (mean 10 years earlier, *p* = 0.035) and lower CI (*p* = 0.049). Finally, this change was more prevalent in IPAH patients (*p* = 0.040). Other clinical and hemodynamic parameters exhibited no statistically significant differences. These results should be analyzed carefully because all carriers for c.572G>A (p.G191D) variation but one, were also carriers for mutations in others genes (*BMPR2*, *ACVRL1* and *KCNA5*).

## Discussion

Mutations in the *ENG* gene have been described in up to 88 % of HHT patients, including some with PAH associated with HHT [29, 30]. In this study we have identified a higher number of pathogenic mutations in comparison with the results showed by other analysis [4, 7, 17, 31–33]. All research conducted in *ENG* gene have been performed in IPAH or HPAH patients, but the study by Pfarr et al [7] described a small number of pathogenic mutations in patients with congenital heart disease associated to PAH. In 29 children with IPAH or HPAH and 11 with APAH due to congenital heart disease without any symptoms or familial history of HHT, Pfarr et al [7] found 2 patients (5 %) carriers of mutations in the *ENG* gene. However, in our cohort we included patients with IPAH and associated with other pathologies. This is the first mutational analysis of the *ENG* gene in PAH patients associated to connective tissue disease, human immunodeficiency virus and porto-pulmonary hypertension.

We identified *ENG* mutations in 16 subjects, a significantly higher percentage. We detected 5 *ENG* mutations with potential pathogenicity not yet described and three described sequence variants. Furthermore, with the in silico analysis we were able to classify synonymous mutations and mutations located in intronic junctions as pathogenic mutations. However, other studies only focused on the analysis of missense and nonsense mutations [7, 32]. Perhaps this fact can significantly increase the percentage of pathogenic mutations in our patients. For these analyses we used eight bioinformatic softwares that analyzed the pathogenicity of the mutations. We considered PolyPhen, Pmut, Sift and Mutation Taster softwares that analyze the amino acid conservation, the protein function or the protein structure [21–24]. However, these softwares show some differences in the criteria used to establish the pathogenicity character of the variation. Some of them included more information as the description of the variants when is possible, the implication in the splicing process or the presence of enhancer sequences. Besides, we used four softwares that analyze the implication of the splicing changes in the mRNA processing. In silico analysis is not totally reliable, and for this reason we believe that analyze this variants with several softwares is necessary to give us a greater approach to catalogue a variant as polymorphism or as pathogenic mutation. Functional studies would be necessary to confirm the pathogenicity of these variants. Aparisi et al [34] described that after exhaustive in silico analysis with splicing softwares, only a few mutations classified as pathogenic resulted really pathogenic in the functional splicing analysis performed. In our study, we have to take into account the fact that none of these variations classified as pathogenic have been found in healthy controls and the c.1633G>A (p.(G545S)) mutation was been classified as pathogenic by other research group [7].

We detected two hot spots for exons 6 and 12 in the *ENG* gene. These exons are located in the extracellular region (Zona pellucida-like domain) [7], a very important area rich in glycosylation sites and cysteine residues [15, 20]. This region has a characteristic pattern of preserved residues [15, 20, 35]. Furthermore, Ali et al have reported that missense mutations in this region for *ENG* gene led to a decrease or disappearance of cell surface expression of the protein [36]. Likewise, many missense mutations located in an orphan domain, situated in a Zona pellucida-like domain, resulted in protein misfolding, altering the subcellular localization [35]. It is likely that the mutated protein was retained by the endoplasmic reticulum (ER) quality control machinery [26, 36]. As a result, the protein becomes trapped in the rough ER and is subjected to ER associated protein decay [26]. Thus, disruption of the downstream signaling of the TGF-β pathway might be caused by mutations affecting both the TGF-β/ALK1 and TGF-β/ALK5 balance and the endothelial-cell growth potential [37–39]. The number and class of molecules involved in this pathway, which differ among cells, underlie the great complexity and versatility of TGF-β signaling [31]. Moreover, in vitro studies on pulmonary artery smooth muscle cells from IPAH patients have indicated growth abnormalities [40].

Missense changes found in these patients are located in aminoacidc residues highly conserved except p.(S432C) and p.(R554C). These variations could be explained as polymorphic change with evolutionary effects. Serine is a non-essential polar amino acid that is neutrally charged, and arginine is non-polar, essential and neutrally charged. However, cysteine is non-essential and negatively charged. The change in charge could be compensated with another mutation in a region in close three-dimensional proximity. Gallione et al [20] reported that cysteine amino acids are involved in disulfide bridging. These mutations can produce alterations in the protein’s structure that affect its functionality; the mutant allele could have a dominant negative effect over the wild type allele, causing serious consequences for carrier patients as have been described by John et al [41] in the *BMPR2* gene in patients with PAH.

The c.572G>A (p.(G191D) change has been previously described as a polymorphism or rare variant [26–28] despite being classified as pathogenic with four of the computer programs used. For this reason and because it is found at a very low frequency in the Spanish and European control population, we performed functional studies for this mutation to verify in vitro its pathogenicity. The analysis with the minigenes assay did not detect any change in the splicing process. Förg T et al [26] performed several colocalization experiments with fluorescence microscopy, and the authors also classified it as a polymorphism. Nonetheless, it is possible that this change may act through other mechanisms, as the complete role of *ENG* is still unknown and requires further functional studies.

Furthermore, we found a pathogenic synonymous change. Synonymous changes could interfere with the splicing accuracy, translation fidelity, mRNA structure and protein folding. Furthermore, these mutations may decrease the half-life of mRNA, leading to downregulation of the protein expression [8, 33]. Synonymous codons are translated at lower levels than standard codons, since specific tRNA levels are decreased [42]. Functional studies for synonymous mutations, intronic changes and intronic duplication would be very interesting, as the role of these changes is unknown, and a functional approach could help us to improve our knowledge of the disease.

In addition, we found that carriers of pathogenic mutations were younger at diagnosis. This fact, together with previous studies, indicates significant heterogeneity in the genetic background of PAH. Mutations in the *BMPR2* gene are most common in PAH patients, but other genes may be related, including *ACVRL1* or *KCNA5* [8]. All patients in this study were analyzed for mutations in these three genes (*BMPR2*, *ACVRL1* and *KCNA5*) [8]. For the 57 patients analyzed for *ENG* gene, 11 out of 16 patients exhibited only a mutation in the *ENG* gene. Mutations in the *ENG* gene are quite prevalent in our cohort of PAH patients, can influence the development of the pathology and did not appear in 55 control samples.

The ability of *ENG* to collaborate in the pathogenesis is highly variable, as described by Mallet et al [43]. The mutant protein could act in a haploinsufficient manner, interacting with the wild type protein and interfering in the normal endoglin function; alternatively, reduction or loss of the cell surface expression of the mutant protein has been described. As noted by John et al [41], we cannot exclude other mechanisms, including the ability to interact with other partners or to activate other signaling pathways.

When we compared the hemodynamic and clinical parameters between patients with and without pathogenic mutations, patients with mutations exhibited a significantly earlier age at diagnosis (8 years compared with patients without mutations) and a lower 6MWT. Therefore, we cannot exclude the possibility that these differences may be due to the small number of patients in our series. PAH exhibits highly variable clinical parameters, and clinical diagnosis is complicated by the heterogeneous outcome of disease manifestation; hence, additional diagnostic tools are required to perform early diagnosis in affected individuals.

Considering the patients with mutations only in the *ENG* gene, we did not find significant differences in clinical or hemodynamic parameters, but patients were diagnosed at an earlier age compare with patients without mutations. Endoglin exhibits two different splice isoforms, short (S) and long (L). Although the most common isoform of endoglin in endothelial cells is L-endoglin, Blanco et al [44] reported that short S-endoglin expression contributes to the cardiovascular pathology associated with age in vivo and in vitro. These results suggest that S-endoglin expression affects the senescent program of endothelial cells when S-endoglin is upregulated instead of being solely responsible for senescence. Furthermore, Liu et al [45] reported that endoglin is also related to critical function in the development of the vascular system in mouse embryonic stem cells, this could explain that patients with pathogenic mutations have an early presentation of the disease.

Previous studies in the *BMPR2* gene indicate that PAH patients carrying a mutation have an onset of disease approximately 10 years earlier than non-carriers [4] and Liu et al [46] suggest that the phenotype of PAH patients with *BMPR2* mutations are influenced by gender. These male patients have a more penetrant phenotype [46]. The former statement of the *BMPR2* gene could be extrapolated to the *ENG* gene, according to our results, but we did not detect gender differences in this study.

As almost all of our patients with the c.572G>A (p.G191D) change exhibited a pathogenic mutation in other genes (*BMPR2*, *ACVRL1* and *KCNA5*), we investigated whether the presence of this change could modify the phenotype. Pfarr et al [7] found significant differences for a low PVR value when they compared carriers of mutations in the *BMPR2*, *ACVRL1*, *ENG* and *SMAD* genes with non-pathogenic mutation carriers. Moreover, we found significant differences in the age at diagnosis, CI and PAH types when comparing hemodynamics and clinical parameters between patients with the c.572G>A (p.G191D) change vs patients without pathogenic mutations in none analyzed genes. Patients harboring this mutation exhibited significantly smaller CI values. We found that this mutation was more prevalent in patients with IPAH than in those APAH. Finally, this mutation appears in patients who are diagnosed 10 years earlier than non-carriers. As the specific mechanism for *ENG* is not yet characterized and its relation with other PAH genes remain unclear, these data should be cautiously interpreted.

Five patients with pathogenic mutations in the *ENG* gene also exhibited a mutation in another gene. Two of these patients with p.(Q166Q) mutation in the *ENG* gene [47] are carriers of p.(Q92L) and p.(V341M) *BMPR2* gene mutations, classified as pathogenic [8]. Patient 3, with a c.1272+6A > T mutation, was also a carrier of the p.(W298*) mutation in the *BMPR2* gene [8]. The last two patients, with p.(G545S) [7] and c.817+17T>A mutations, also harbored the p.(S232T) and p.(T243N) *ACVRL1* gene mutations, respectively. Mallet et al [43] described several patients with pathogenic mutations in different genes, including *ENG,* in HHT patients. The authors proposed that one of the two mutations classified as pathogenic could be a rare variant [43], unlikely to cause PAH. However, as observed in other human pathologies, oligogenic inheritance of PAH with a major causal gene should not be excluded [48]. Rodríguez-Viales *et al* [49] proposed that additional variations can produce a more severe phenotype and an early disease. The evaluation of the total mutation load could be the way to understand how mutations in different genes could be responsible for the disease [50, 51]. This fact further supports the importance of the functional analysis of these mutants.

The low number of patients included in this study represents a handicap to our ability to draw stronger conclusions, but the comprehensive investigation and complete follow-up of all cases adds value to our data. The high clinical evaluation of these patients and the expertise in the molecular methodology field make us confident about the elevated presence of putative pathogenic mutations in these patients. A functional study should be necessary in order to corroborate the functional impact of these mutations.

## Conclusion

To conclude, we described mutations in the *ENG* gene in IPAH and APAH patients, some of which have not been previously described. The vast majority of the mutations found in this study are private, making difficult to establish a correlation between the phenotype and a particular mutation. For this reason, the genotype-phenotype correlation was performed according to all mutations found in a group of patients. Mutations in the *ENG* gene may influence the clinical status of the disease. Moreover, genetic analysis in patients with PAH may be of clinical relevance and demonstrates the complexity of the genetic background. A better understanding of the molecular basis will support the future design of individualized treatments according to the genetic background of each patient.

## Abbreviations

6MWT, 6 min walking test; ACVRL1, activin A Receptor Type II-Like 1; APAH, associated pulmonary arterial hypertension; BLAST, basic local alignment search tool; BMPR2, bone morphogenetic protein type 2 receptor; CAEI, Comité Autonómico de Ética da Investigación; CI, cardiac index; CT, tomography computerized; ENG, Endoglin; ER, endoplasmic reticulum; ERS-ESC, European Respiratory Society-European Society of Cardiology; FC, functional class; HHT, hereditary hemorrhagic telangiectasia; HIV, human immunodeficiency virus; HPAH, heritable pulmonary arterial hypertension; H-WE, Hardy-Weinberg Equilibrium; IPAH, idiopathic pulmonary arterial hypertension; KCNA5, potassium voltage-gated channel, shaker-related subfamily, member 5; L: long; mPaP, mean pulmonary artery pressure; mRNA, messenger RNA; PAH, pulmonary arterial hypertension; PCR, polymerase chain reaction; PVR, pulmonary vascular resistance; S, short; SD, standard deviation; sPaP, systolic pulmonary artery pressure; TGF-β, transforming growth factor beta.
